# Effects of Ni/Co doping on structural and electronic properties of 122 and 112 families of Eu based iron pnictides

**DOI:** 10.1038/s41598-023-40419-8

**Published:** 2023-08-12

**Authors:** Joanna Stępień, Damian Rybicki, Marcin Sikora, Zbigniew Bukowski, Michał Babij, Łukasz Gondek, Czesław Kapusta, Tomasz Strączek, Kamil Goc, Danilo Oliveira De Souza

**Affiliations:** 1 Academic Centre for Materials and Nanotechnology, AGH University of Science and Technology, al. A. Mickiewicza 30, 30-059 Kraków, Poland; 2 Faculty of Physics and Applied Computer Science, AGH University of Science and Technology, al. A. Mickiewicza 30, 30-059 Kraków, Poland; 3 Institute of Low Temperature and Structure Research, Polish Academy of Sciences, ul. Okólna 2, 50-422 Wrocław, Poland; 4 National Synchrotron Radiation Centre SOLARIS, Jagiellonian University, Czerwone Maki 98, 30-392 Kraków, Poland; 5 ELETTRA Sincrotrone Trieste S.C.p.A., S.S. 14 Km 163.5, 34149 Trieste, Italy

**Keywords:** Superconducting properties and materials, Electronic properties and materials

## Abstract

Superconductivity in high-temperature superconductors such as cuprates or iron pnictides is typically achieved by hole or electron doping and it is of great interest to understand how doping affects their properties leading to superconductivity. To study it we conducted Fe and As *K* edge x-ray absorption spectroscopy measurements on several electron doped compounds from the 112 and 122 family of Eu-based iron pnictides. XANES and EXAFS results confirm that dopants are located at expected sites. For both families we found an electron charge redistribution between As and Fe occurring with doping. The changes it caused are stronger in the 112 family and they are bigger at As sites, which indicates that doped charges are predominantly localized on the dopant site. However, the results obtained do not provide clues why Ni doping in 122 family does not lead to occurrence of superconductivity.

## Introduction

One of the biggest challenges in physics is the understanding of the high-temperature superconductivity, which typically occurs when holes or electrons are doped to a magnetically ordered undoped “parent compound”. Chemical doping both in iron-pnictides and cuprates raises a question where the doped charge is localized and how it changes electronic properties of materials leading to superconductivity^1,2^. Iron pnictides are a family with the second highest $$T_c$$ values after the cuprates and there are already many families of iron pnictides known. One of the most intensively studied is the 122 family (*A*Fe$$_2$$As$$_2$$, where $$A = $$ Ca, Sr, Ba, Eu, etc.). In this family superconductivity can be induced by mechanical pressure, isovalent substitution and by electron or hole doping on Fe or As sites.

Another family, which does not receive so much scientific attention, is the 112 family with its first discovered member Ca$_{1-x}$La$_x$FeAs$_2$ with $T_c$ up to 35 K^3^. The most notable difference between 122 and 112 families is the crystal structure. In 122 families there are two alternating layers: of *A* and Fe-As while in the 112 family every second Fe-As layer is replaced by As in zig-zag chains. Recently, Eu based 112 family has been successfully prepared with superconductivity achieved by La doping in Eu$_{1-x}$La$_x$FeAs$_2$^4^. Unlike in Ca$_{1-x}$La$_x$FeAs$_2$, the stoichiometric parent compound EuFeAs$_2$ can be prepared and, interestingly, Eu magnetic moments order at two times higher temperature (40 K) compared to the 122 family. Possibility to prepare the parent compound allows to conduct studies starting from the undoped compound, which potentially can result in better understading of chemical doping effects. Soon after, superconductivity has been successfully achieved by Ni doping at the Fe site with maximum $T_c$ of 17 K^5^. This is in contrast to the 122 Eu(Fe$_{1-x}$Ni$_x$)$_2$As$_2$ system, which is not superconducting at all^6^. It has been shown recently that substitution of Fe with Co in the 112 family leads to superconductivity as well with maximum $T_c$ value up to 28 K^7,8^, which is two times higher than in the 122 Co doped family. Here, we show results of Fe and As *K* edge x-ray absorption spectroscopy measurements on several superconducting and not superconducting compounds from the 112 and 122 family, which were conducted with the aim to study the effect of Ni and Co doping on local electronic and crystal structure at Fe and As sites and to compare it between members of both families.

## Experimental

The samples studied belong to two families of iron pnictide superconductors i.e. 122 family with general formula Eu(Fe$_{1-x}$TM$_x$)$_2$As$_2$ and 112 family EuFe$_{1-x}$TM$_x$As$_2$, where TM is Ni or Co. In the case of the 122 family there was also one sample where Eu was partially replaced by Sr. All samples from the 122 were prepared as single crystals and their preparation and characterization are described in the literature^9^. Samples from the 112 family were prepared as polycrystalline powders and their preparation method and characterization can be found in literature^8^. More details regarding the samples, including the doping and transition temperatures are provided in Table 1. In the case of Co doped samples we selected samples from 112 and 122 families, which have similar $T_c$ values while for Ni doping we selected samples with similar Ni content since Ni doping in 122 family does not result in superconductivity.

**Table 1:** List of samples studied including parent compounds (122-u and 112-u) and doped ones.

Family	Symbol	Formula	T$_{SDW}$ (Fe) [K]	T$_{Eu}$ [K]	T$_c$ [K]
122	122-u	EuFe$_2$As$_2$	190	19.9	–
122	122-Ni3	Eu(Fe$_{0.965}$Ni$_{0.035}$)$_2$As$_2$	124	16.3	–
122	122-Co15	Eu(Fe$_{0.85}$Co$_{0.15}$)$_2$As$_2$	117	16.0	7.2
122	122-Sr50Co12	Eu$_{0.5}$Sr$_{0.5}$(Fe$_{0.88}$Co$_{0.12}$)$_2$As$_2$	–	10.0	15.0
112	112-u	EuFeAs$_2$	100	40.1	–
112	112-Ni4	EuFe$_{0.96}$Ni$_{0.04}$As$_2$	–	37.5	20.4
112	112-Co2	EuFe$_{0.98}$Co$_{0.02}$As$_2$	93	40.0	4.0
112	112-Co5	EuFe$_{0.95}$Co$_{0.05}$As$_2$	84	39.8	12.0
112	112-Co8	EuFe$_{0.92}$Co$_{0.08}$As$_2$	–	39.4	27.5

Values of the spin density wave transition temperature (T$_{SDW}$) of Fe magnetic moments, ordering temperature of Eu magnetic moments (T$_{Eu}$) and critical temperature of superconductivity (T$_c$) of superconducting samples are also provided.

XAS measurements at *K* edge of Fe and As were performed at XAFS beamline^10^ of Elettra Sincrotrone Trieste, Italy. XAFS beamline is installed on a bending magnet source and covers energy range from 2.4 to 27 keV with resolving power $E/\Delta E$ equal to 10$^{-4}$ (for Si 111) or 10$^{-5}$ (for Si 311). Powder samples were mixed with BN and pressed into pellets to obtain optimum absorption length for transmission detection. Two sets of samples were prepared to optimize absorption length for both Fe and As. All XAS measurements were carried out at room temperature since previous measurements on similar compounds showed almost no temperature dependence of spectra, see e.g. Ref.^11^. Data analysis was done using Demeter package^12^.

Simulations of *K* edge XANES were performed using FDMNES^13,14^. They were done as a self-consistent calculations in the multiple scattering mode on a cluster with the radius of 6.5 Å. Doping was simulated by substituting one of the Fe sites in the nearest neighbourhood of the absorbing atom with Co or Ni, without taking into account structural relaxations (rigid structure simulations). In the 112 family, where 3 inequivalent As lattice sites exist, the simulated As *K* edge spectrum was averaged (with proper weights) over calculated spectra of all the inequivalent sites. Gaussian convolution at Fe *K* edge was 2.0 eV, while Fermi energy cutoff was -3.0 eV for the 122 family and -1.3 eV for the 112 family. Gaussian convolution at As *K* edge was 2.4 eV, while Fermi energy cutoff was -3 eV for both families.

## Results and discussion

**Figure 1:**
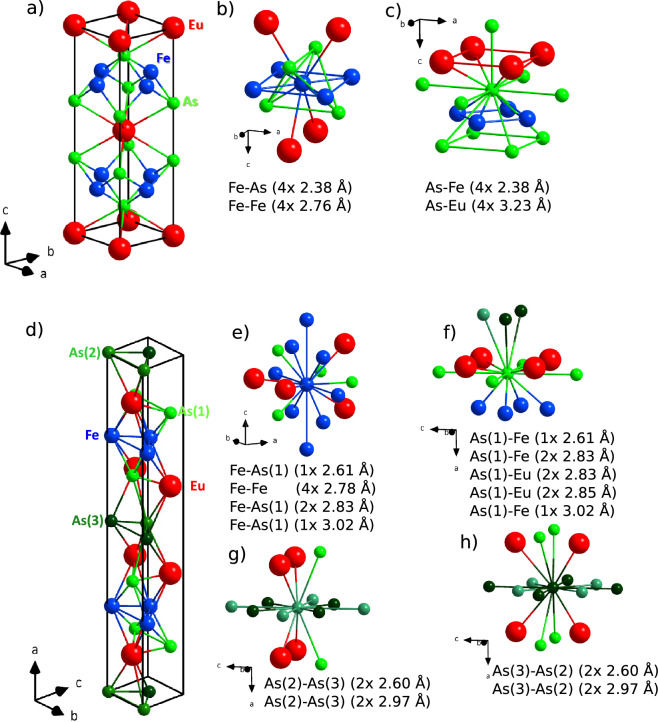
(**a**) Room temperature crystal structure of the undoped compound from the 122 family with local atomic surrounding of Fe (**b**) and As (**c**), and of undoped compound from the 112 family (d) with local atomic surrounding of Fe (**e**) and As(1) (**f**), As(2) (**g**) and As(3) (**h**). Distances are from our XRD measurements and are provided for first two coordination shells or up to 3 Å, multiplicities of atoms are provided as well.

Before we proceed to XAS results we briefly discuss features of crystal structures derived from XRD^8,9,15^. In Fig. 1 we show unit cell and local environments of Fe and As for undoped compounds from the 122 and 112 family. At room temperature the crystal structure of 122 compounds can be indexed by the tetragonal *I*4/*mmm* space group (No. 139)^9,15^. In the unit cell there is only one type of Eu, Fe and As site with Eu atoms lying in a plane separated by As-Fe layers. On a closer look As atoms form corners of tetrahedra with Fe located inside of it. Compounds from the 112 family have the crystal structure belonging to the orthorhombic *Imm*2 space group (No. 44)^8^. In this family one Fe is missing in the formula unit compared to the 122 family. Due to this in every second layer, which separates Eu planes, there are only As atoms forming so called zig-zag chains. As a result there are three As sites: As(1) located in the As-Fe layer, As(2) and As(3) in zig-zag chains. In the As-Fe layer one can still identify As tetrahedra, although they are strongly distorted compared to almost ideal ones present in the 122 family.

### XANES on Fe and As *K* edges

**Figure 2:**
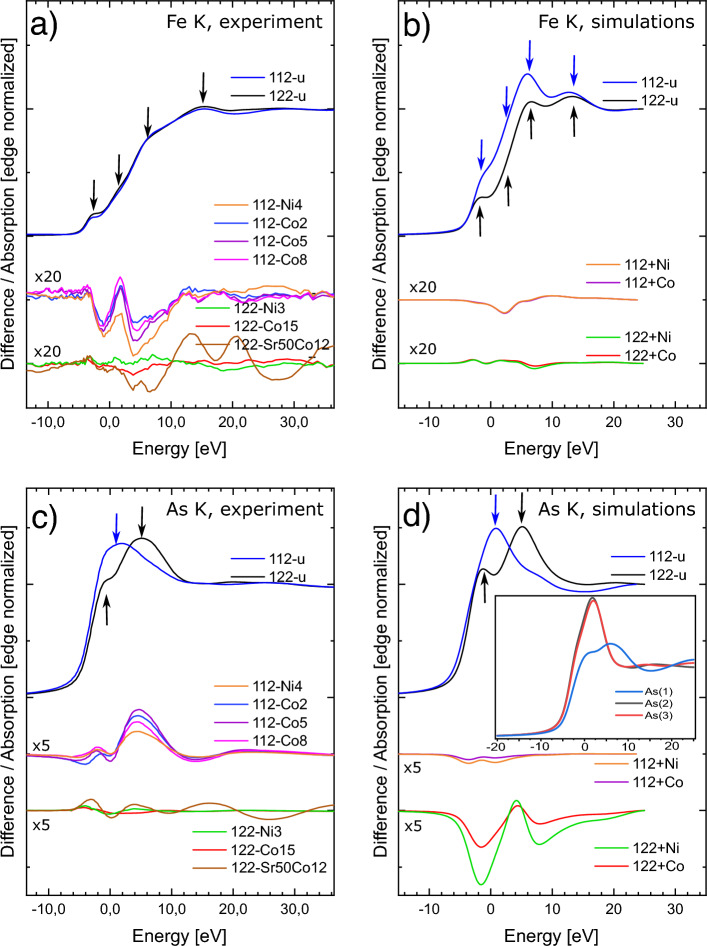
Fe and As *K* edge XANES spectra. (**a**) Experimental Fe *K* edge spectra of undoped compound from 112 and 122 family and differences between spectra of doped and undoped compounds from both families. (**b**) Simulated Fe *K* edge spectra of undoped compounds and differences between spectra calculated with Ni/Co doping, zero energy corresponds to 7115.5 eV (**c**) Experimental As *K* edge spectra of undoped compounds and differences as in (**a**). (**d**) Simulated As *K* edge spectra of undoped compounds and differences between spectra calculated with Ni/Co doping, inset shows simulated spectra for all three As sites, zero energy corresponds to 11868.6 eV.

We start the discussion of XAS results with Fe *K* near edge spectra, which can be divided into two parts. The first is the main absorption edge, which is due to transitions from the 1*s* core state to the 4*p* continuum. The second is the pre-peak region which is usually attributed to quadrupole transitions from the 1*s* core state to the empty 3*d* states and is ascribed to the hybridization between Fe 3*d* and As 4*p* orbitals^16,17^. As such the pre-peak intensity may be related to the modification of the local electronic density of the absorbing element, while the main edge structure can be considered as a fingerprint of modification of the local structural environment, which can be caused by chemical doping. For all samples from both families pre-peak is located at about 7113 eV and three features at the absorption edge at about 7117 eV, 7120 eV and 7130 eV can be noticed (see Fig. 2a). The spectrum of the undoped compound from the 122 family is similar to already reported one^18^ and to that of a similar compound BaFe$_2$As$_2$^11,17,19,20^. If one compares the spectra of undoped compounds from 112 and 122 families (Fig. 2a) one can see that that there are some minor differences e.g. intensity of the pre-peak and at vicinity of the main peak (7130-7140 eV), but the overall shape and edge energy are very similar. This is in contrast to theoretical simulations of Fe *K* edge based on structural data obtained from XRD. The simulations (Fig. 2b) show significant differences between 122 and 112 undoped compounds, which is expected considering their dissimilar Fe atomic environments (Fig. 1). We note that energy positions of all main features in simulated spectra for both families are almost identical, but their relative intensities differ. The simulated spectra for the 122 family is close to the experimental one, while in the case of the 112 family the first peak in the simulated white line is stronger. This is due to difference in the more distant neighbour shells and the scattering on them, which is accounted for in the simulation performed within the radius of 6.5 Å. Experimental data of both families show close similarity indicating negligible influence of more distant neighbours on photo electron scattering and the electronic structure at Fe.

Another observation is that Co/Ni doping (electron doping) in the case of both families has only a minor influence on the shape of the Fe *K* edge spectra. This has been observed e.g. Co doped BaFe$_2$As$_2$ and it was concluded that Co does not charge dope the Fe ions^19^. However, when BaFe$_2$As$_2$ was hole doped either by K doping of Ba site or Cr doping of Fe site there were significant changes in the intensity of the pre-peak while the absorption edge energy remained unchanged^17^. Our simulations (Fig. 2b) indicate that the influence of charges doped into rigid crystal structure is modest. Therefore, to better visualize the effect of doping on spectral shape, we show a difference between the spectra of doped and undoped samples in both, experiment and simulations. The difference in Fe *K* edge absorption spectra of 112 family are very similar for both, Co and Ni. The spectral intensity of doped samples is expected to be lower in the region of raising edge (single minimum) and higher in the range just above white line (weak maximum), Fig. 2b. In the experimental spectra we observe stronger differences caused by doping with two minima, (Fig. 2a). The first one, which is weakly dependent on doping type and level, is located at the pre-peak range (at about 7114 eV), while the other one is located at the energy range of the first edge feature (at about 7120 eV) and reveals intensity dependent on doping. The simulated difference in Fe *K* edge absorption spectra of 122 family is more structured, but weaker than in 112 family and with stronger difference in the case of Ni doping in the energy range corresponding to white line. In the experimental spectra we observe similarly small amplitude of difference spectra as expected by simulations. For Co doping the shape of experimental difference spectra resembles that of simulations, while for Ni doping the minimum expected near white line (at about 7120 eV) is absent. For almost all doped samples the negative difference signal in Fig. 2a indicates a decrease in the effective density of unoccupied *p*-like electronic states at Fe. This effect is weaker for 122 family, especially for the Ni doped sample where it is barely visible. It may be expected considering low amount of Ni dopant. In Fig. 2a we also present experimental results collected for 122-Sr50Co12 sample, which is doped both with Co at Fe site and Sr at Eu site. This is not charge doping since both Eu and Sr are 2+ ions and additionally have very similar ionic radius. Difference spectrum of this sample shows the strongest variations among all the samples of 122 family with strong differences present also well above the white line.

We now move to As *K* edge XANES with experimental spectra shown in Fig. 2c and simulations performed within rigid structure model shown in Fig. 2d. In contrast to Fe *K* edge of the undoped 112 and 122 compounds, their As *K* edge spectra differ significantly (Fig. 2c). For the 122 family one can notice two features in As *K* edge XANES, which are located at about 11868 eV and at about 11874 eV. This is similar to that observed for BaFe$_2$As$_2$^20^. However, in our case the first peak is less pronounced and the second peak is less structured, which is likely due to lower spectral resolution. In the case of 112 family the spectrum seems less structured. It consists of a single, broad maximum centered at about 11870 eV. This is related to presence of two different As structural sites (Fe-As layer and As zig-zag chains) in the 112 family compared to only one in the 122 family (Fig. 1). Our theoretical simulations are in good agreement with experiment. In the the case of 112 family the presence of different lattice sites of As leads to weighted spectral contributions of all the sites, which are shown in the inset of Fig. 2d revealing a strong difference between the spectra obtained for As site located in Fe-As layer, As(1), and As in the zig-zag chains, As(2) and As(3). One can notice that the spectrum of As(1) site is similar to that obtained for the 122 family, which is expected since the local environment of As is similar in both cases. Our simulations of the spectra of undoped samples qualitatively agree with calculations reported by Ghosh et al.^21^. Simulation of the influence of charges doped into rigid crystal structure reveals a striking difference between both families (Fig. 2d). In the case of 112 family additional charge provided by dopants should have much weaker influence on spectral shape compared to 122 family. In both families the spectral changes expected from simulations are proportional to additional charge, namely these are approx. twice stronger for Ni doping with respect to Co. The expected shape of difference spectra is qualitatively similar (two minima) for both families, but the spectral range is different. For the 112 family the differences in charge distribution are expected to affect the region on the rising and decreasing slope of the white line, while for the 122 family the minima are observed at first peak and decreasing slope of the second peak. In the experimental As *K* edge spectra (Fig. 2c) one can see that effect of doping is opposite than predicted by simulations, i.e. it is stronger for 112 family and its sign is reversed. Moreover, the differences observed for spectra of Ni doped samples are weaker than that of Co ones. Similarly to Fe *K* edge XANES, also at As *K* edge the experimental spectrum of 122-Sr50Co12 sample shows the strongest variations among all the samples of 122 family and the strong differences are present also well above the white line. It is worth noting that the experimental difference spectra of As show positive sign, which denotes a decrease of the valence electron density at the As site in contrast to the effect at Fe, where we observed an increase of the electronic population.

In conclusion of XANES measurements we note that both simulated and experimental effects of electron doping (Ni/Co) of the Fe site in 112 and 122 families are rather small, which is similar to results reported for other 122 families. We note that doping may cause structural relaxations, which can have an opposite effect on the spectral shape to that caused by distribution of electronic charges. It is difficult to analyse both effects quantitatively, especially when they are small as in our case. It is expected that charge distribution changes should be visible predominantly on the closest neighbours (i.e. at As), while structural relaxations should be present both on As and Fe. Our rigid structure simulation indicate solely the expected spectral evolution caused by charge distribution changes. Both simulated and experimental changes in spectral shape induced by doping are significantly stronger on As than on Fe, which is anticipated if As is closer to dopant site. This confirms that Co and Ni substitute predominantly Fe. For almost all compounds we observed an increase of the electron population at Fe site with its simultaneous decrease at As site, which indicates a redistribution of electronic charge caused by the doping. Similar effects were reported by Balédent et al., for BaFe$_2$As$_2$ where spectral differences at As upon Co doping were also positive and larger than at Fe^11,19,22^. Doping effects on experimentally measured Fe and As *K*-edge XANES are bigger in the case of the 112 family, which indicates that structural relaxations due to Fe site doping are stronger in 122 family. This may result in higher values of $T_c$ for 112 family, but we admit that it could be partly caused by possible presence of impurities, which were found in traceable amount after synthesis of 112 samples^8^.

Simulated doping induced changes in As *K*-edge spectra of 112 family are much weaker than in the case of 122 family, while for Fe *K*-edge they are of similar amplitude which indicates that changes in charge distribution due to doping at Fe sites are similar in both families. In the 122 family Ni doping has almost the same, small effect on experimental Fe and As *K*-edge spectra as Co doping, but Co containing sample is superconducting while Ni doped is not. It is worth noting that Sr doping into the 122 family has bigger influence on spectral shape of XANES, which can be attributed to structural distortion of the local atomic environment of Fe and As due to lattice relaxation, confer EXAFS spectra below. This confirms previous findings that doping on the *A* site (Eu, Ba etc.) results in stronger spectral changes than at the Fe site. Since Sr doping is not charge doping, it underlines the small effects of electron doping of Fe site in the 122 family.

### EXAFS on Fe and As *K* edges

**Figure 3:**
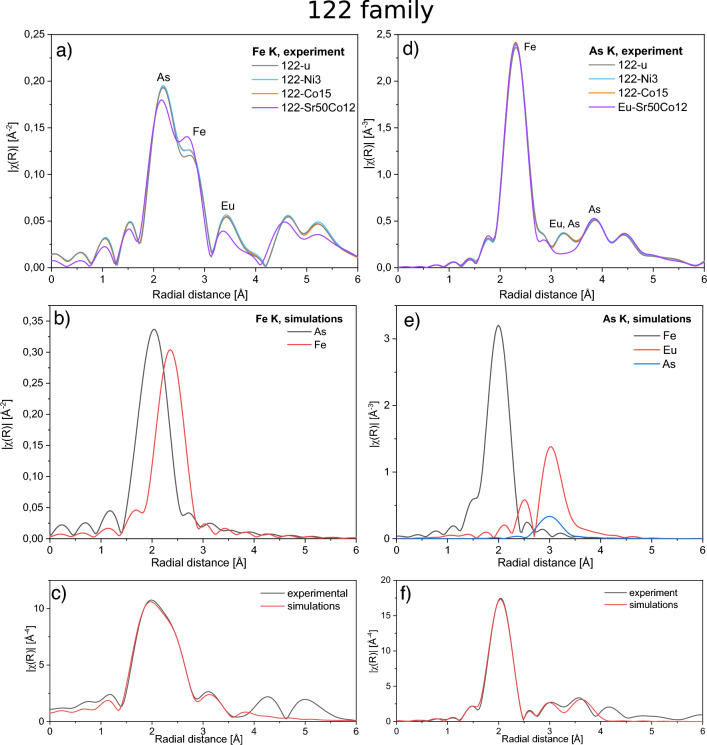
Fe *K* and As *K* EXAFS results on the 122 family. (**a**) Experimental results for Fe *K* EXAFS on undoped and doped compounds. (**b**) Theoretical shape of scattering paths contributing to the first coordination shell of Fe for the undoped compound. (**c**) The best fit of the first shell of Fe for the undoped compound (fit parameters are provided in Table 2). (**d**) Experimental results for As *K* EXAFS on undoped and doped compounds. (**e**) Theoretical shape of scattering paths contributing to the first and second coordination shell of As for the undoped compound. (**f**) The best fit of the first and second shell of As for the undoped compound (fit parameters are provided in Table 2).

**Figure 4:**
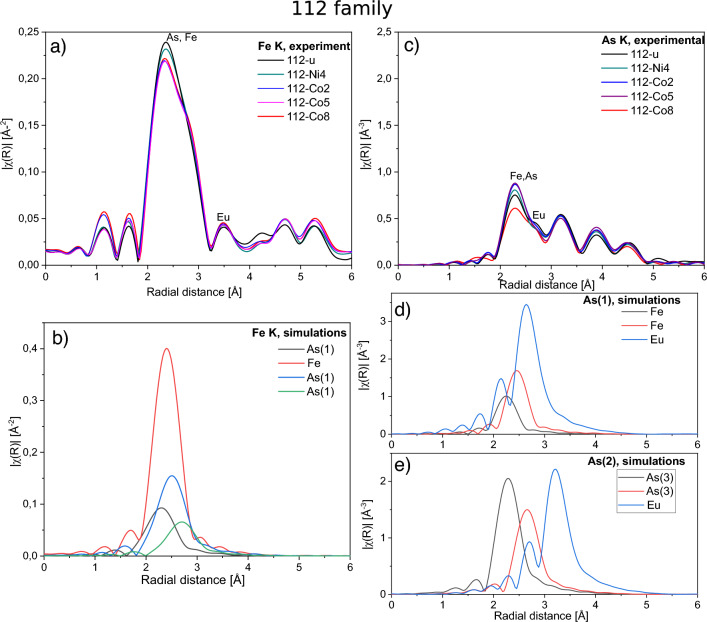
Fe *K* and As *K* EXAFS results on the 112 family. (**a**) Experimental results for Fe *K* EXAFS on undoped and doped compounds. (**b**) Theoretical shape of scattering paths contributing to the first coordination shell of Fe for the undoped compound. (**c**) Experimental results for As *K* EXAFS on undoped and doped compounds. (**d**) Theoretical shape of scattering paths contributing to the first and second coordination shell of As(1) site and (**e**) As(2) site for the undoped compound. Shapes for As(3) are almost identical to those of As(2).

Compared to XRD, EXAFS measurements can provide information not only on the local structure and inter-atomic distances, but also on the local disorder introduced by chemical doping, which is often of interest as disorder can affect electronic properties of materials including superconductivity. We start with EXAFS results on the 122 family, which are shown in Fig. 3. In the case of the local surrounding of Fe in the first coordination shell there are 4 As atoms, in the second shell 4 Fe atoms, and Eu atoms in the third shell, which are all clearly visible in Fig. 1b. We observe changes in the intensity as a function of Ni/Co doping only in the second shell indicating that Co/Ni substitute Fe (Fig. 3a). The situation is different in the case of 122-Sr50Co12, for which there is a decrease of the scattering intensity in the range of the first coordination shell (As) and an increase in the second shell (Fe). For this sample as a result of the fitting we obtained a few percent increase of $\sigma ^2$ (the mean-squared deviation from the mean inter-atomic distance *R*), and decrease of *R* for the first coordination zone compared to Co doped sample (122-Co15), see Table 2. This could be understood by the fact that in the first coordination shell there are As, which are close to Eu doped by Sr. In the case of the second shell the distance *R* is the same, but unexpectedly $\sigma ^2$ is noticeably smaller suggesting better order of atoms in the second coordination shell (Fe) of 122-Sr50Co12 sample.

**Table 2:** Parameters obtained from EXAFS fits for the 122 family including: *R* - the mean interatomic distance, $\sigma ^2$ - the mean-squared deviation from *R* due to the thermal motion and static disorder, Fe-As-Fe angle and *h* - the height of As above the Fe plane.

	122-u			122-Ni3			122-Co15			122-Sr50Co12		
	Fe-As	Fe-Fe	As-Fe	Fe-As	Fe-Fe	As-Fe	Fe-As	Fe-Fe	As-Fe	Fe-As	Fe-Fe	As-Fe
*R* (Å)	2.38	2.74	2.39	2.38	2.75	2.38	2.38	2.75	2.38	2.37	2.75	2.38
$\sigma ^2$ ($10^{-3}$ Å$^{2}$ )	5.6	13.3	5.6	5.5	13.3	5.6	5.6	13.2	5.5	5.9	11.8	5.5
Fe-As-Fe angle ($^\mathrm{{o}}$)	109.29			109.57			109.58			109.97		
*h* (Å)	1.38			1.37			1.37			1.36		

Parameters for Fe-As and Fe-Fe are from Fe *K* edge, and As-Fe from As *K* edge data.

Looking from As atoms (Fig. 3d–f), their first coordination shell in the 122 family consists of four Fe, Fig. 1c. EXAFS intensity of corresponding peak slightly increases with Ni/Co doping as expected from small increase of back-scattering by elements with higher effective atomic number *Z*. There is no noticeable change of $\sigma ^2$ parameter. The second coordination shell is occupied by Eu and As atoms and there are no observable changes for this shell. In contrast to that in sample with Sr substituting Eu there is a strong decrease of the intensity related to significant decrease of the effective *Z*, which confirms that Sr really substitutes Eu. Overall, Co/Ni doping does not result in significant changes of *R* or $\sigma ^2$ in the first two coordination shells indicating negligible impact on local structure. Such a behavior was also observed in Co doped BaFe$_2$As$_2$^23^. Only Sr doping appears to cause small increase of disorder in the first shell (Fe-As) and decrease in the second shell (Fe-Fe), which is opposite to effect of potassium doping in Ba$_{1-x}$K$_x$Fe$_2$As$_2$^24^.

The crystal structure of samples from the 112 family has much lower symmetry, which strongly affects the local surrounding of Fe and As atoms (Fig. 1d–h) with three nonequivalent As lattice sites. Looking from Fe, already in its nearest environment (FeAs$_4$ tetrahedron) there are three different distances to arsenic atoms in As(1) site. This unfortunately makes fitting of the EXAFS experimental data not feasible as there would be to many fit parameters and, therefore, we can only discuss qualitatively the experimental results shown in Fig. 4. The intensity of the first coordination shell of Fe (As(1)) decreases with doping indicating an increase of local disorder. The intensity of the second coordination shell (Fe atoms) slightly increases with substituting Fe with Co/Ni i.e. dopants with higher *Z*. This indicates that order of the second shell is preserved. EXAFS on As indicates that with Co/Ni doping there are strong changes of the intensity mostly in the first coordination shell, which involves As(1)-Fe and As(1)-As(1). These strong changes are in qualitative agreement with EXAFS measurements on Fe and are due to an increase of the local disorder with doping. Disorder could be caused by movement of As(1) in the structure or, more likely, could result from presence of impurity phases observed by XRD, which amount is found to increase with doping^7,8^.

From experimental observations it has been proposed that the highest $T_c$ values in iron pnictides are obtained for compounds with the most regular FeAs$_4$ tetrahedra^25,26^ i.e. with the Fe-As-Fe angle close to 109.47$^\mathrm{{o}}$ and with optimal height of As above the Fe plane, *h* of about 1.38 Å.^27^ We note that in all samples from the 122 family the Fe-As-Fe angle and As height are very close to optimal ones (Table 2). Values, which are the most different from optimal are for Sr doped sample 122-Sr50Co12, which has the highest $T_c$ (15 K). It appears that it is due to weakening of the Eu magnetism, which must thus be stronger than structural considerations, since the maximum $T_c$ achieved by Co doping of EuFe$_2$As$_2$ is of about 10 K^9^ while of SrFe$_2$As$_2$ maximum $T_c$ is 20 K^28^. Interestingly, values of the angle and height in not superconducting 122-Ni3 and superconducting 122-Co15 samples are almost identical. The amount of Ni with respect to Fe in 122-Ni3 sample corresponds to that giving superconductivity in the 112 family and in Ca based 122 family, Ni doping of CaFe$_2$As$_2$ gives maximum $T_c$ of about 15 K^29^. Similar impact of Ni and Co doping on EXAFS shape indicates that local structural properties seem not to be responsible for the lack of superconductivity in the case of Ni doping in Eu based 122 family. A recent study by some of us showed that it also not related to Eu magnetism^30^ as was suggested earlier^6^ and lack of superconductivity upon Ni doping of EuFe$_2$As$_2$ remains an open question.

In the 112 family, which has a lower crystal symmetry, FeAs$_4$ tetrahedra are very strongly distorted and yet Co doping of EuFeAs$_2$ results in higher maximum $T_c$ value of about 28 K^7^ compared to 10 K in the 122 family. Even more striking is presence of superconductivity in Ni doped EuFeAs$_2$ with maximum $T_c$ of about 18 K^31^. The biggest difference between 112 and 122 families is the strongly distorted first coordination shell of Fe in 112 family. EXAFS spectra show that it is also more sensitive to doping than in the 122 family.

## Conclusions

Our XANES and EXAFS results on Eu based 112 and 122 families of iron pnictides confirm that dopants are located at expected sites, i.e. Co/Ni replaces Fe and Sr replaces Eu. EXAFS indicates that Co/Ni doping has small effect on the local crystal structure in the 122 family while in the 112 changes due to doping are stronger and they are mostly related to As(1) sites, i.e. those in the first coordination shell of Fe. XANES measurements and rigid structure simulations show that influence of Co/Ni doping on the local charge distribution is also rather small, but for almost all compounds we observed an increase of the electron population at Fe site with its simultaneous decrease at As site, which indicates a redistribution of electronic charge caused by the doping. The changes induced by doping are more pronounced in the case of the 112 family and they are bigger at As sites, which indicates that additional charges are predominantly localized on the dopant site. Interestingly, both EXAFS and XANES on the 122 family show that Ni doping has very similar effects to Co doping but Co containing sample is superconducting while Ni doped is not, which suggests that local crystal and charge environment do not support superconductivity in the case of Ni doping to EuFe$_2$As$_2$.

## Data Availability

The datasets used and/or analysed during the current study are available from the corresponding author on reasonable request.
